# Delayed onset of hypertrophic cardiomyopathy in a 61-year-old male patient with *MYBPC3* mutation

**DOI:** 10.1186/s44348-024-00009-2

**Published:** 2024-08-12

**Authors:** Suyon Chang, Hoon Seok Kim, Mi-Hyang Jung, Myungshin Kim, Jong-Chan Youn

**Affiliations:** 1 Department of Radiology, Seoul St. Mary’s Hospital, College of Medicine, The Catholic University of Korea, Seoul, Korea; 2 Department of Laboratory Medicine, Seoul St. Mary’s Hospital, College of Medicine, The Catholic University of Korea, Seoul, Korea; 3 Division of Cardiology, Department of Internal Medicine, Seoul St. Mary’s Hospital, Catholic Research Institute for Intractable Cardiovascular Disease (CRID), College of Medicine, The Catholic University of Korea, 222 Banpo-daero, Seocho-gu, Seoul, 06591 Korea

**Fig. 1:**
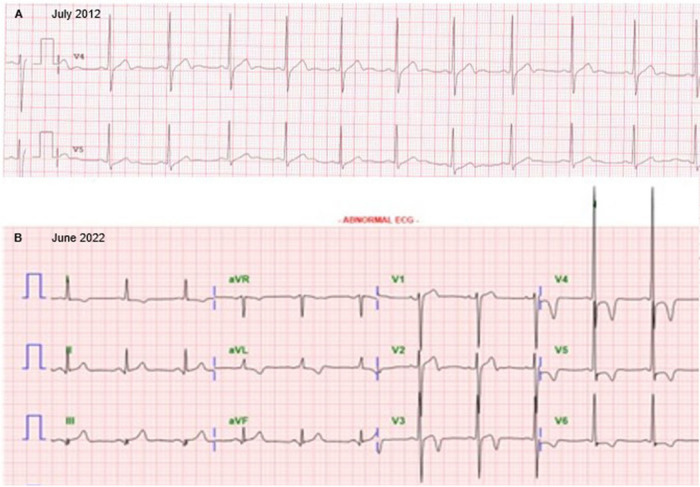
Serial electrocardiography at baseline and 10-year follow-up. Electrocardiography at baseline (**A**) and 10-year follow-up (**B**)

**Fig. 2:**
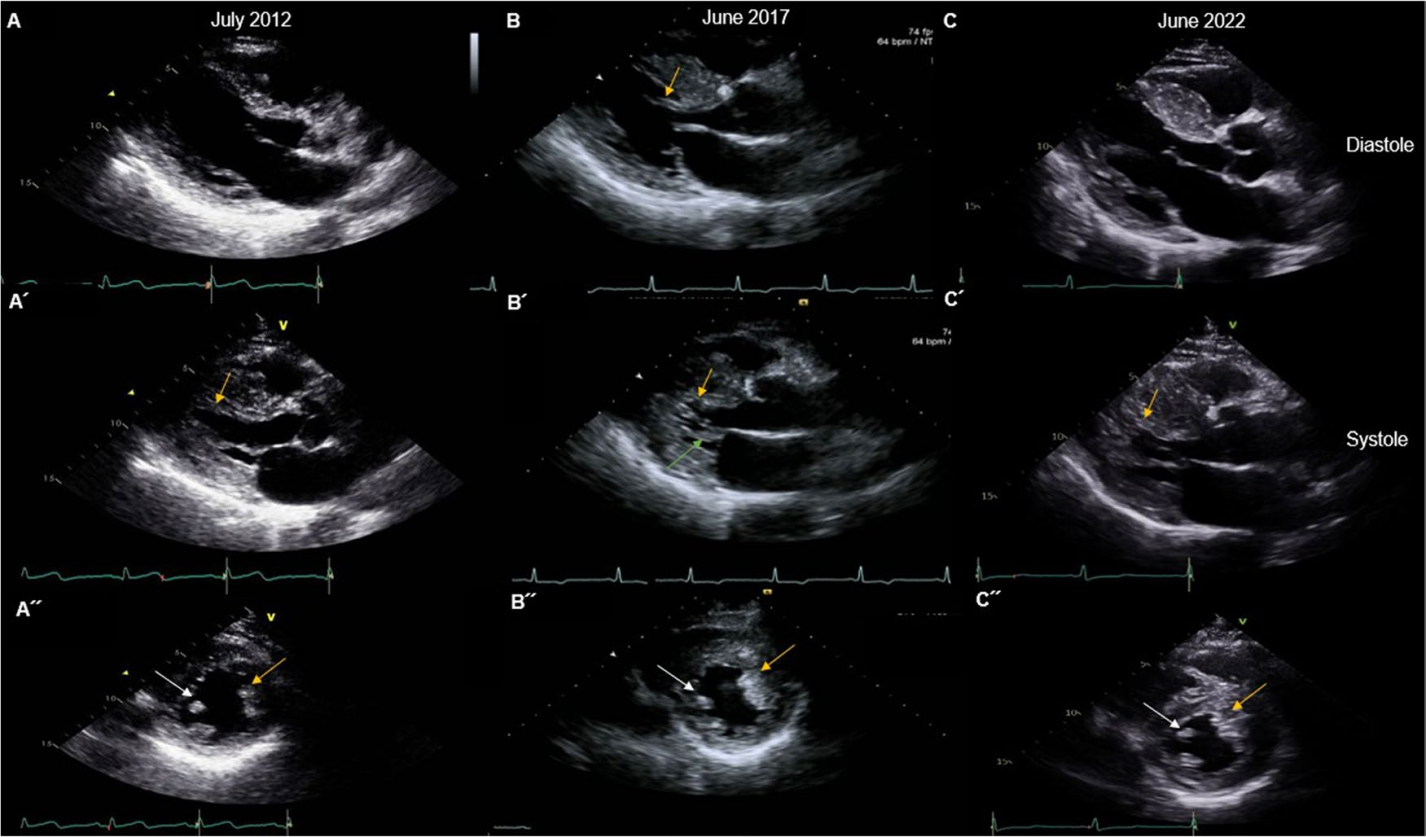
Serial echocardiography at baseline, 5-year, and 10-year follow-up. Echocardiography revealed non-obstructive hypertrophic cardiomyopathy with left ventricular hypertrophy that had gradually progressed over 10 years. The left (**A**), middle (**B**), and right (**C**) panels indicate echocardiographic images at baseline (July 2012), 5-year follow-up (June 2017), and 10-year follow-up (June 2022), respectively. The upper and middle panels are parasternal long-axis echocardiographic images acquired at diastole and systole. Lower panels are images for the parasternal short axis at the papillary muscle level

**Fig. 3:**
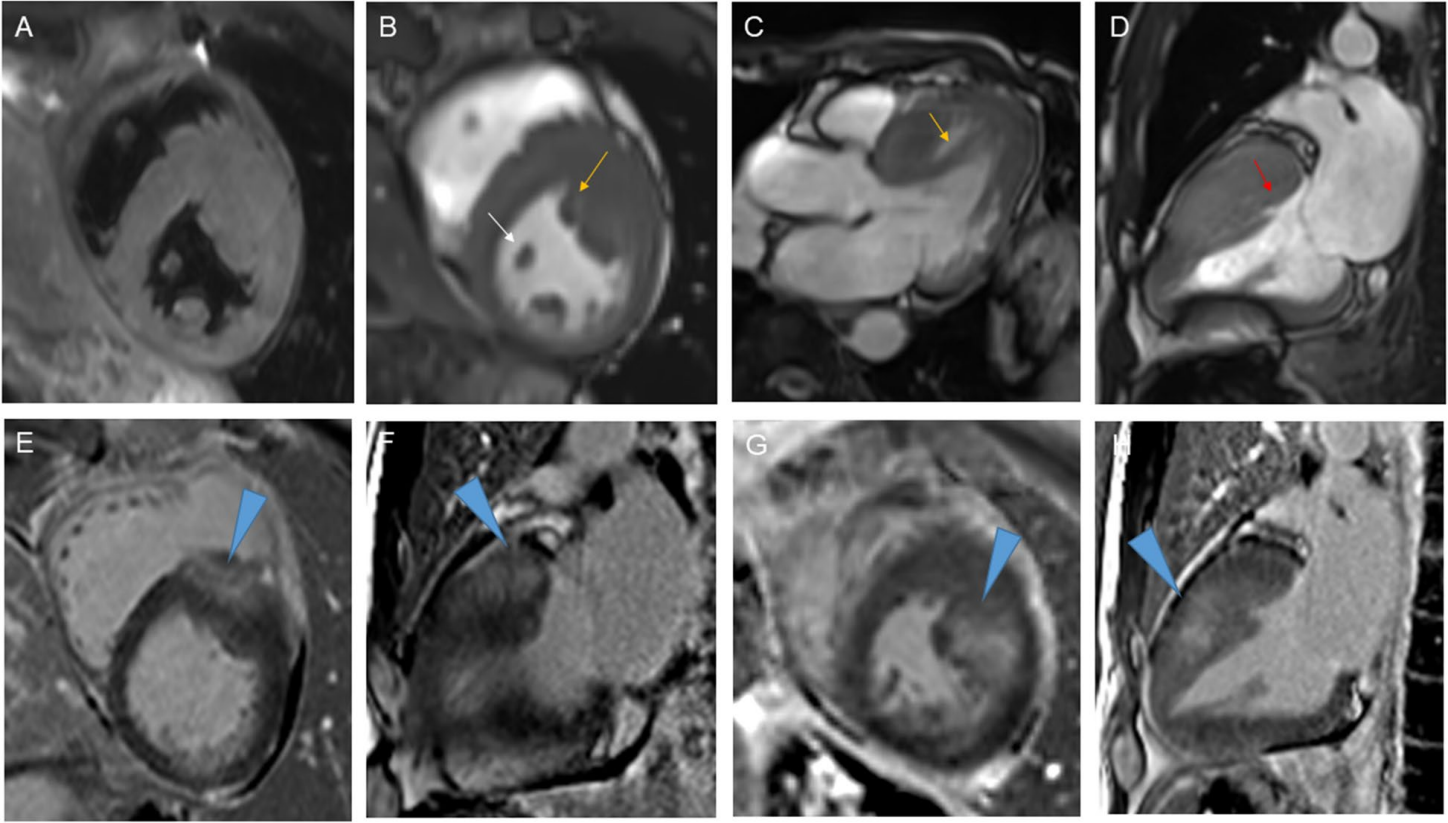
Cardiac magnetic resonance images acquired at the 10-year follow-up. T2-weighted image in short-axis orientation (**A**) shows the thickening of the anteroseptal wall and papillary muscle of LV. Cine images in short-axis (**B**), 3 chamber (**C**), and vertical long-axis (**D**) orientation demonstrate an accessory apical-basal muscle bundle inserting into the basal anteroseptum (yellow arrow), anomalous tendinous connection to the septum (red arrow), and another accessory papillary muscle (white arrow). Late gadolinium enhancement images in basal (**E** and **F**) and mid (**G** and **H**) LV show patchy enhancement in the thickened myocardium and papillary muscles (blue arrowheads). LV: left ventricle

**Fig. 4:**
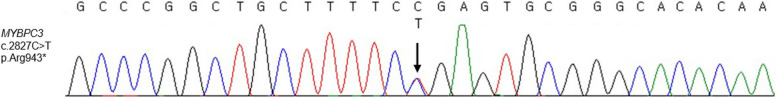
DNA sequencing chromatogram of the patient. The direct sequencing showed a heterozygous nonsense mutation (black arrow), NM_000256.3:c.2827C>T (p.Arg943*), in exon 27 of *MYBPC3* gene

A 61-year-old man with controlled hypertension visited the clinic for intermittent dizziness. Holter monitoring revealed no significant pause or arrhythmia. Echocardiography showed asymmetrical septal hypertrophy, but no evidence of dynamic left ventricular (LV) outflow tract obstruction, highly suggestive of non-obstructive type hypertrophic cardiomyopathy (HCM). Intriguingly, LV hypertrophy gradually progressed over 10 years. The electrocardiogram had changed from normal to LV hypertrophy (Fig. 1). Serial echocardiography demonstrated an increased septal wall thickness from 11.1, 13.3 to 17.8 mm (Fig. 2, Movies 1, 2 and 3). Potential clues suggestive of HCM in earlier images were anomalous papillary muscle direct insertion to the septum (yellow arrows) and mitral leaflet (green arrow), and accessory papillary muscle (white arrows). These features were confirmed with cardiac magnetic resonance images acquired at the 10-year follow-up (Fig. 3). Although he did not have a familiar history of HCM, clinical exome sequencing revealed a heterozygous pathogenic mutation of *MYBPC3*, NM_000256.3:c.2827C>T (p.Arg943*), subsequently validated through direct sequencing (Fig. 4, black arrow). This study describes the natural course of delayed onset hereditary HCM. In most HCM, LV hypertrophy develops during childhood and adolescence; thus, LV wall thickness does not change once early adulthood is reached. However, as in our cases, delayed onset HCM is recently being recognized [1, 2]. *MYBPC3* or *MYH7* are the most commonly found gene mutations in hereditary HCM. A prior study reported a milder prognosis and delayed disease onset in patients with *MYBPC3* mutation than in *MYH7* mutations, as in our case [1]. Early suspicion and meticulous familial screening, counseling, and genetic tests might help manage HCM.

### Supplementary Information

**Additional file 1:** **Movie 1.** Initial echocardiographic images at July 2012 (parasternal long axis view).

**Additional file 2:** **Movie 2.** Five-year follow-up echocardiographic images at June 2017 (parasternal long axis view).

**Additional file 3:** **Movie 3.** Ten-year follow-up echocardiographic images at June 2022 (parasternal long axis view).
